# The critical role of injecting drug users on the spatial distribution of hepatitis C virus; a study in the West of Iran 

**Published:** 2018

**Authors:** Farid Azizi Jalilian, Masoud Parvin, Meysam Olfatifar, Hossein Erfani, Jalal Bathaei

**Affiliations:** 1 *Virology Department, Faculty of Medicine, Hamadan University of Medical Sciences, Hamadan, Iran*; 2 *Student Research Committee, Hamadan University of Medical Sciences, Hamadan, Iran*; 3 *Gastroenterology and Liver Diseases Research Center, Research Institute for Gastroenterology and Liver Diseases, Shahid Beheshti University of Medical Sciences, Tehran, Iran*; 4 *Deputy of Health, Hamadan University of Medical Sciences, Hamadan, Iran *

**Keywords:** Hepatitis C virus, Spatial analysis, Injecting drug users, Hamadan province

## Abstract

**Aim:**

This study was conducted to provide a clear epidemiological picture of HCV spatial pattern.

**Background:**

Hepatitis C virus (HCV) is one of the major problems of public health, that its spatial and spatiotemporal pattern remain unclear in Hamadan province.

**Methods:**

We used the scan statistic to identify the spatial and spatiotemporal clusters of HCV in Hamadan province with an emphasis on considering the role of carrier's and injecting drug users (IDUs) cases. We repeated the same analysis to estimate the effect of some influencing factors on the formation of clusters. All HCV cases that had been recorded by deputy health of Hamadan University of Medical Sciences during 2008-2016 were included in this study.

**Results:**

The location of the purely spatial cluster for carriers, IDUs and total of cases were similar to each other, a cluster consisting of Toyserkan, Nahavand, Asadabad, Malayer and Bahar cities. However, after adjustment, the location of the identified cluster for both carries and IDUs cases changed to a cluster consisting of Asadabad, Bahar, Toyserkan and Nahavand cities. This cluster also observed for spatiotemporal clusters carriers, IDUs and total of cases even after adjustment.

**Conclusion:**

Although further studies in individual level are needed, our results revealed that spatial distribution of HCV in Hamadan province (especially in clusters areas) can strongly dependent on the distribution of IDUs cases. Consequently, the effectiveness of HCV combating programs is subjected to properly controlling these case through various counseling, behavioral and therapeutic programs.

## Introduction

 Hepatitis C virus (HCV) is known as the main cause of chronic liver diseases, cirrhosis and even hepatocellular carcinoma (1) both in developed and developing countries(2) So that it is the third cause of cancer-related death in the worldwide and its burden will be increased in the future years. although the overall prevalence of HCV in Iran is probably less than one per cent (3). Controlling and prevention of HCV due to lack of vaccination and injecting drug users (IDUs), blood and infected blood products, its high costs (4, 5) and contaminated medical and non-medical instruments transmission way is a public health problem not only in Iran but also in many other countries(6) and its projected that HCV to be the most important leading cause of viral hepatitis-related mortality in Iran (7, 8).

So that, increase of injecting drug users (IDU) cases have challenged the elimination of HCV despite the decrease in the prevalence of HCV regarding mandatory screening programs of blood donations(9). Therefore, to better deal with HCV changes in health policies are needed (9), and from the WHO guidelines for response to HCV are raising awareness, promotion partnership, mobilizing resources, formulation of evidence-based guidelines and executive information (10). 

To accomplish this task, one of the way is spatial analysis that can provide a solid framework to enhance our ability to study and monitor of diseases(11) in term of the surrounding context, transmission patterns and assessment of prevention interventions(12), and also can be served as the way for conducting more molecular and serological epidemiology to accurately explain of the disease burden. The objective of the present study is to examine the HCV clusters in Hamadan province. It is hoped that the science of the great impact of IDUs cases on the formation of clusters in this province led to the adoption of appropriate measures to respond to HCV. 

## Methods

**Study setting**

 Hamadan province is one of the western cities of Iran with an area of 19493 square kilometers. Hamadan province had a population of 1758268 people; according to the national census held in 2011 by Statistical Center of Iran (https://www.amar.org.ir/english). It has 9 cities entitled Asadabad, Bahar, Hamadan (center of Hamadan province), Famenain, Kabudrahang, Malayer, Nahavand, Toyserkan, and Razan.

**Study population**

The HCV data were obtained from the health deputy of Hamadan University of Medical Sciences from Jan2008 to Dec 2016 that in each case includes the age, sex, occupation, transmission method, marital status, residence (rural/urban) and date of diagnosis.

The Hamadan province population data were downloaded from Iranian national statistic center(13) and in combination with HCV cases data were used to building the SaTScan software (version 9.6) requirement files (by default to run Poisson distribution software needs to three files including cases, population and geographical coordinates), Geographic Information System (GIS) version 10.3 was also used to building the coordinate file.

**Spatial analysis**

Poisson distribution was used to explore the purely spatial and spatiotemporal clusters spatial scan statistic with discrete. Clusters detection software impose a cylinder window to the map that its height shows the time and its radius represent the space (Obviously for purely spatial cluster only a circle imposed on map) so that is placed on assigned coordinates center of each block (city) and in each case calculate the maximum likelihood ratio using below equity and compared to amount calculated based on Monte Carlo simulation to test null hypothesis (constant risk over space and time, inside and outside of cluster).

$I={\left(\frac{c}{\mathrm{EI}(c)}\right)}^{c}{\left(\frac{C-c}{C-E(c)}\right)}^{C-c}$

In this formula C is the total number of cases, c is observed cases inside the window, E (c) is expected cases inside the window, C-c and C-E [c] are observed and expected cases outside of search window respectively. Moreover, in order to more exactly explain the epidemiology of HCV, the same analysis was repeated for carrier and IDUs cases. Clusters were adjusted for age, sex, occupation, and marital status and residence (urban/rural) variables. 

**Table 1:** High rates purely spatial clusters of hepatitis patients in Hamadan province, both for unadjusted and adjusted status

Clusters ID	Locations	No. of cases	Expected cases	Relative risk	Log likelihood ratio
Primary clusters					
Total of cases					
Adjusted	To, Na, As, Ma, Ba^*^	697	522.69	1.91	55.74
Unadjusted	To, Na, As, Ma, Ba	697	463.79	2.37	99.91
Carrier cases					
Adjusted	AS, Ba, To, Na	166	89.14	2.06	30.09
Unadjusted	To, Na, As, Ma, Ba	609	427.02	2.31	74.43
IDUs cases					
Adjusted	AS, Ba, To, Na	112	53.78	2.32	27.06
Unadjusted	To, Na, As, Ma, Ba	418	292.73	2.34	51.94

^*^ Na: Nahavand; As: Asadabad; Ma: Malayer; Ba: Bahar; To: Toyserkan

**Figure 1:**
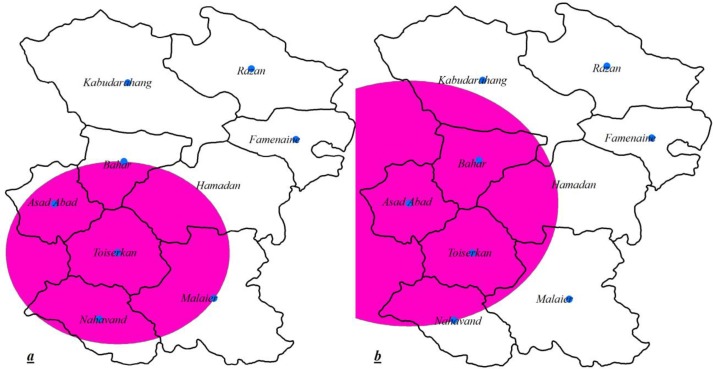
Spatial analysis of HCV cases in Hamadan province; (a) high rates purely spatial cluster of HCV; (b) high rates spatiotemporal cluster of HCV and (whites color areas aren’t significant)

**Table 2:** High rates spatiotemporal clusters of hepatitis patients in Hamadan province both for unadjusted and adjusted status

Clusters ID	Locations	Time period	No of cases	Expected cases	Relative risk	Log likelihood ratio
Total of cases						
Primary clusters						
Adjusted	As, Ba, To, Na	2010*2014	119	56.67	2.19	26.71
Unadjusted	As, Ba, To, Na	2010*2014	119	42.60	3.01	48.67
Carrier cases						
Primary clusters						
Adjusted	As, Ba, To, Na	2009*2013	95	48.71	2.06	14.88
Unadjusted	As, Ba, To, Na	2010*2014	97	36.74	2.84	36.06
IDU cases						
Primary clusters						
Adjusted	As, Ba, To, Na	2010*2014	66	30.53	2.3	16.51
Unadjusted	As, Ba, To, Na	2010*2014	66	24.41	2.91	25.55

^*^ Na: Nahavand; As: Asadabad; Ba: Bahar; To: Toyserkan

## Results

**Basic Information**

During eight years (2008 to 2016), 1100 cases of HCV have been reported, which of them 986 (0.88%) were male and 132 (0.12%) were female, 884 (80.36%) lived in urban areas and 216 (19.64%) at rural areas subsequently. The mean age was 42.4±13.8 years. The most of the cases were married (67.55%), not vaccinated (*82.55*) and injecting drug users’ cases (55.91%).

**Spatial analysis**

*Spatial clusters*

patial analysis can provide essential information regarding the high risk geographic areas and populations of diseases that can lead to better management of diseases.. Therefore, we applied the scan statistic method in this study. Results indicated that same primary cluster was detected even after adjustment for age, sex, occupation, and marital status and residence variables. This cluster with the centrality of Toyserkan city and a radius of 49.05 km had been formed of the Toyserkan, Nahavand, Asadabad, Malayer and Bahar cities (Table1& Fig.1a), but adjustment shifted the location of carrier and IDUs cases clusters that were same to total cases clusters, a clusters with centrality of Asadabad city and radius of 64.99 km had been composed of Asadabad, Bahar, Toyserkan, and Nahavand (Table 1& Figure 1b). Suggesting that the majority of HCV in Hamadan province are the carrier and injecting drug users that probably have markedly effect on the formation of detected clusters.

*Spatiotemporal clusters*

Considering the importance of detecting clusters, we also explored the existence of spatiotemporal clusters. The location of detected clusters for all cases, carrier and IDUs cases were similar to each other even after adjustment and with the centrality of Asadabad city and radius of 64.99 km had been composed from the Asadabad, Bahar, Toyserkan and Nahavand cities (Table2&Fig1.b)(same as adjusted purely spatial clusters of carrier and IDUs cases). Except, for the adjusted cluster of carriers cases the time period of other detected clusters was between 2010 and 2014 (Table2). However, the characteristics of detected clusters had some differences from each other (Table2). These results can also indicate the substantiality effect of the carrier and injection drug users on the formation of clusters.

## Discussion

Our finding emphasizes the need to implementation the more strictly intervention against the carrier and injecting drug users cases to the proper response to HCV in Hamadan province. We have identified that these cases have the greatest impact on the formation of detected clusters. So that, the location of spatiotemporal clusters of total, carrier and IDUs cases were constant even after adjustment. This cluster with the centrality of Asadabad city and radius of 64.99 km had been composed of the Asadabad, Bahar, Toyserkan and Nahavand cities (**Table 3**&**Figure b**) and was similar to purely spatial adjusted clusters of carrier and IDUs cases(Table2&**3**). In addition, the small number of cases attributed to carrier and IDUs clusters can appropriately justify the major role of these cases information of detected clusters. 

In keeping with other studies(14), IDUs cases had more likely to be infected with HCV and longer duration of IDUs was related with more HCV prevalence. Other evidence has suggested the need to adaption of the treatment setting to integrate of the IDUs cases in HCV treatment(15). In addition, another study (16) stated that the epidemiology of HCV in Iran has changed and due to the development of IDUs cases has increased. Furthermore, the most of simulation study (17) consider the IDUs cases as a prerequisite for the elimination of HCV to 2030(18). HCV prevalence at 52.2% among IDUs group compared to general population (0.3%) and other high-risk groups such as peoples with health-related exposures (0.20%) and population with liver-related condition (7.5%) in Iran can properly justify the aforementioned evidence.

 Likewise, being injection drug user and history of the prison were the most common high-risk behaviours in Hamadan province(19) so that, 38% of IDU prisoners in Hamadan province are HCV Ab positive that is much more than non-IDUs prisoners (20). Other evidence indicated that the HCV, HBV (hepatitis B virus) and HIV (human immunodeficiency virus) markers and their combination were common among IDUs cases(21). One of the possible limitations of this study was under-reporting of HCV cases registered in surveillance system due to its subclinical nature of diseases so that Poorolajal *et al. *(3) also have pointed to it and stated that reliance on surveillance system reports cannot be very true. Another limitation of this study used the circular scan statistic to detect the clusters. However the strengthen of our study was formulating the geographic distribution of HCV cases in Hamadan province.

We can probably propose an integrating confronting strategy against the IDUs related of HCV in Hamadan province regarding a cluster with centrality of Asadabad city and radius of 64.99 km had been composed of Asadabad, Bahar, Toyserkan, and Nahavand (Table2& Figure 1b). In such way our finding can be served as a framework to implement coherent actions and conduct future Sero-epidemiological and molecular studies against IDUs cases because probably other factors in addition to mentioned variables can affect the formation and properties of detected clusters.
